# N-glycosylation of GDF15 abolishes its inhibitory effect on EGFR in AR inhibitor-resistant prostate cancer cells

**DOI:** 10.1038/s41419-022-05090-3

**Published:** 2022-07-19

**Authors:** Rong Wang, Piaopiao Wen, Ganglong Yang, Yanyan Feng, Yuanyuan Mi, Xiaoying Wang, Shenglong Zhu, Yong Q. Chen

**Affiliations:** 1 Wuxi School of Medicine, Jiangnan University, Wuxi, Jiangsu Province 214122 China; 2 School of Food Science and Technology, Jiangnan University, Wuxi, Jiangsu Province 214122 China; 3 School of Biological Engineering, Jiangnan University, Wuxi, Jiangsu Province 214122 China; 4 Department of Urology, Affiliated Hospital of Jiangnan University, Wuxi, Jiangsu Province 214122 China

**Keywords:** Prostate cancer, Prognostic markers

## Abstract

Castration-resistance of prostate cancer is one of the most challenging clinical problems. In the present study, we have performed proteomics and glycomics using LNCaP model. Growth differentiation factor-15 (GDF15) level is increased in androgen receptor (AR) inhibitor-resistant cells and the inhibitory effect of GDF15 on epithelial growth factor receptor (EGFR) pathway is relieved by GDF15 N70 glycosylation. Interference of GDF15 (siRNA or N70Q dominant negative) or EGFR pathway (inhibitor or siRNA for EGFR, SRC or ERK) decreases the resistant-cell survival in culture and tumor growth in mice. Our study reveals a novel regulatory mechanism of prostate cancer AR inhibitor resistance, raises the possibility of AR/SRC dual-targeting of castration-resistance of prostate cancer, and lays foundation for the future development of selective inhibitors of GDF15 glycosylation.

## Introduction

Androgen deprivation therapy (ADT) is a common practice for the treatment of advanced prostate cancer (PCa) since its discovery by Huggins and Hodges in 1941 [1], which involves surgical castration such as bilateral orchiectomy or chemical castration such as inhibition of androgen receptor (AR). This treatment is initially effective, however, nearly all patients progress to castration-resistant prostate cancer (CRPC) [2]. Despite significant efforts [3], CRPC challenge is largely unmet.

Classical pathways of castration resistance have been studied extensively [4, 5]. Additional mechanisms are being explored using omics approach, for instance genomics [6–8], methylomics [9], transcriptomics [10, 11], proteomics [10, 12], and glycomics [13, 14]. Although both “AR-independent” and “AR-bypass” scenario have been described, androgen signaling pathway plays a critical role in CRPC. Dual-targeting tactic for CRPC is worthy of exploring.

Growth differentiation factor-15 (GDF15)/macrophage inhibitory cytokine-1 (MIC-1) is a member of the TGF-β superfamily [15]. GDF15 can suppress food intake and inflammation and thus is a potential candidate to treat many metabolic diseases [16]. GDF15 may also play a part in cancer development and progression depending upon cancer type, stage, and microenvironment [17]. It has been shown that GDF15 blocks norepinephrine-induced myocardial hypertrophy via inhibition of EGFR transactivation [18]. EGFR is reported to be involved in CRPC [19–21], however, it is unclear why clinical trials show insignificant benefit in CRPC patients with EGFR inhibitors [22–24].

Here, we have performed proteomics and glycomics in LNCaP culture model, discovered an important role of GDF15-EGFR pathway in the development of AR inhibitor resistance, and demonstrated its therapeutic potential. Our study may draw research interests in GDF15 and clinical efforts on CRPC using SRC/AR dual-targeting approach.

## Results

### AR inhibitor-resistant prostate cancer cell model is established

**Fig. 1:**
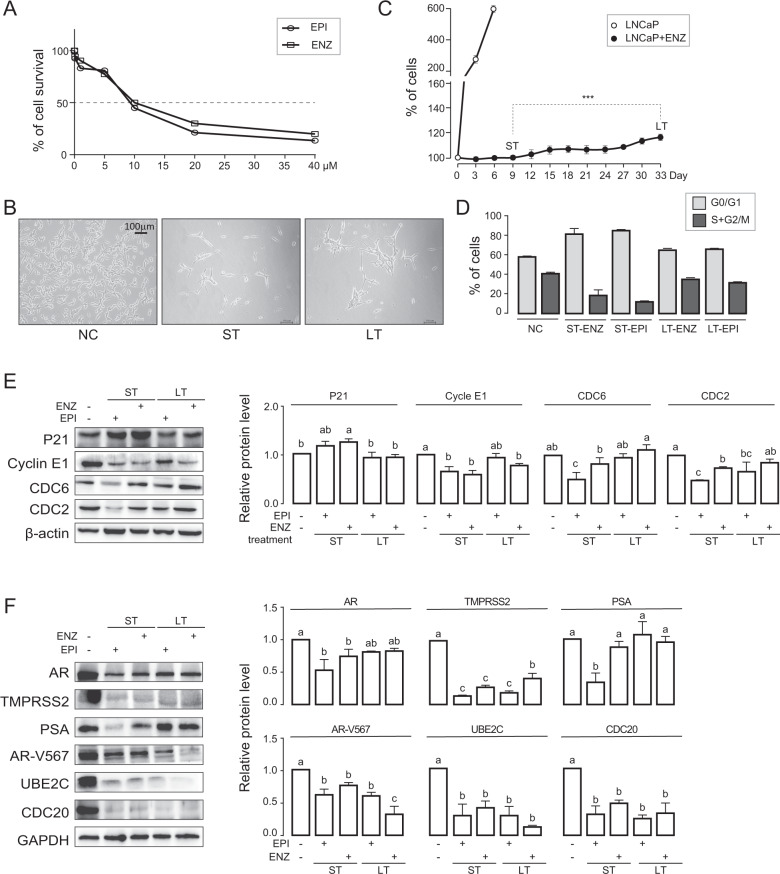
AR inhibitor-resistant prostate cancer cell model is established. **A** 6*10^3^ cells were plated in 96-well plates and treated with various concentrations of ENZ and EPI for 48 h. The survival curve was detected by CCK8. **B**, **C** 1*10^6^ LNCaP cells were plated and either untreated (negative control; NC), treated with ENZ (10 µM) or EPI (8 µM) with normal FBS media change every 3 days for a total of 9 days (short-term treatment, ST) and for 33 days (long-term treatment, LT). Representative microscopic images (**B**) and cell proliferation curve (**C**) are shown. Data were expressed as mean ± std. of biological triplicates; Student’s *t*-test were performed; *p < 0.05. **D** Cells from NC, ST-EPI, ST-ENZ, LT-EPI, and LT-ENZ were analyzed for cell cycle distribution by FACS. **E** Levels of cell cycle markers are quantified in NC, ST, or LT cells by WB. β-actin protein is used as loading control. One-way ANOVA with Turkey test, *p* < 0.05. Data are expressed as mean ± std of biological triplicates. **F** Levels of AR, AR target (TMPRS2, PSA), AR-V567, AR-Vs target (UBE2C, CDC20) proteins in NC, ST-EPI, ST-ENZ, LT-EPI, and LT-ENZ were quantified by WB. GAPDH protein was used as loading control. One-way ANOVA with Turkey test, *p* < 0.05. Data are expressed as mean ± std of biological triplicates.

To establish AR inhibitor-resistant prostate cancer (IRPC) cell model, we treated LNCaP prostate cancer cells with enzalutamide (ENZ) and EPI-001 (EPI) for 9 days (short-term or ST) and for 33 days (long-term or LT), at concentrations of 10 µM (ENZ) and 8 µM (EPI), respectively, based on IC_50_ values of cell survival rates in normal FBS culture medium (Fig. 1A), which were used in all drug-treatment experiments. As an androgen-deprived medium condition, we found the IC_50_ values of DMSO + ENZ and DMSO + EPI in culture medium of charcoal-stripped FBS (c-FBS) were 0.12 µM and 0.07 µM, respectively. Furthermore, with the artificial synthetic androgen methyltrienolone (R1881) manually added in c-FBS cultured medium, the IC_50_ values of R1881 + ENZ and R1881 + EPI were 8.5 µM and 10 µM (Figure S1A), respectively. The results indicated that compared to the absence of androgen in culture medium, a higher concentrations of AR inhibitors were required to inhibit cell growth in the presence of androgen (either manually added or raw contained androgen in culture mediums). At the same time, cell proliferation was completely inhibited during the ST period and the cell slowly regained some proliferative capacity during the LT period, which indicated the treated cells acquired drug-resistance in LT (Fig. 1B, C). The similar phenomenon was observed in the cells with androgen-deprived culture medium (added the same doses of DMSO) or in the manually added R1881 culture medium (Figure S1B, C).

We next examined markers of cell cycle checkpoint by Western Blot (WB) and cell cycle distribution by fluorescence activated cell sorter (FACS) analysis in ST and LT cells. WB results revealed that P21 (G1 phase marker, cyclin-dependent kinase inhibitor) was upregulated in ST and backed down in LT cells, and Cyclin E1 (G1-S phase marker), CDC6 (G1-S phase marker), and CDC2 (G1-S and G2-M phase marker) were downregulated in ST and up again in LT cells (Fig. 1E). Concomitantly, we found G0/G1 arrest in ST which was recovered in LT cells (Fig. 1D).

Further, we determined ENZ/EPI effects on AR and its targets (PSA, TMPRSS2), AR-Vs, and its targets (UBE2C, CDC20). WB results showed that all markers were downregulated to various degrees in ST and LT cells compared to NC (Fig. 1F) with exception of PSA in LT cells. The androgen-deprived (with DMSO condition) and androgen manually added (with R1881 condition) experiments in RNA level also indicated similar results for above-mentioned targets, and the growth markers including C-MYC, AKT1 were downregulated in ST and up again in LT, which was consistent with the above cell proliferation and cell cycle results (Figure S1D-E). These results confirm the effectiveness of ENZ/EPI on the AR pathway at least in the ST cells. Nest, our attention focused on discovering the vital pathway(s) that regulated the cell re-growth in IRPC and exploring the underlying mechanisms.

### GDF15 is identified as a major glycoprotein in IRPC

**Fig. 2:**
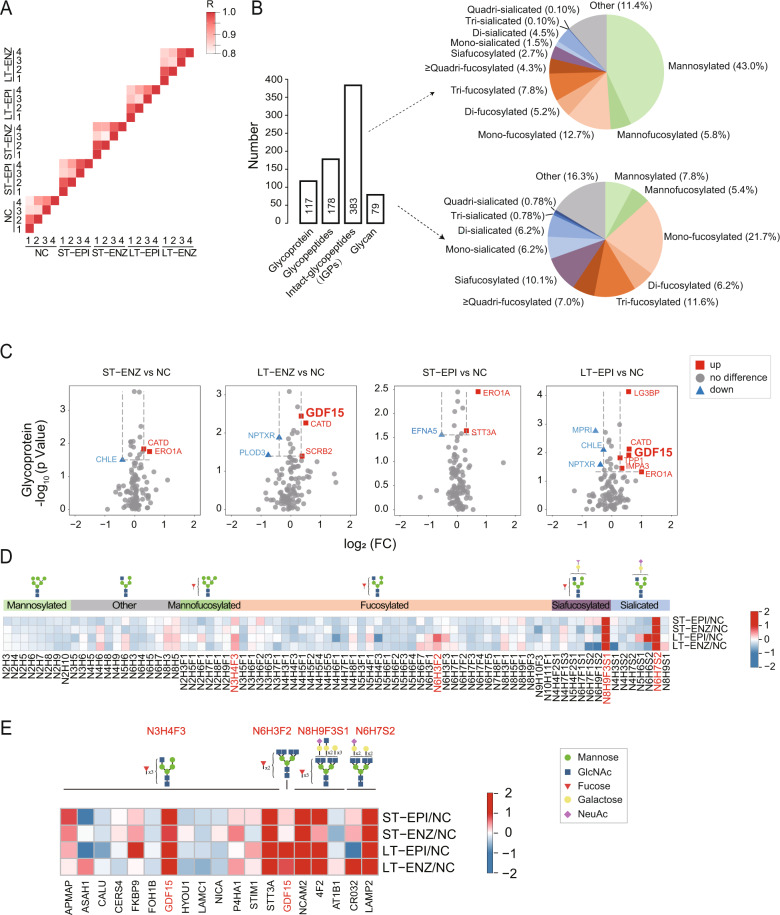
GDF15 is identified as a major glycoprotein in IRPC. **A** Pearson correlation coefficient heatmap of quadruplicates indicates reliability of experiment. **B** Numbers of glycoproteins, glycopeptides, intact *N*-glycopeptides (IGPs), and glycans identified are presented. **C** Volcano plots indicate differentially expressed glycoproteins. *p* < 0.05. **D** Heatmap shows differentially expressed glycotypes. **E** Heatmap displays proteins modified by up-regulated glycotypes. Red indicates up-regulation, blue indicates down-regulation, and gray indicates no difference. N is HexNAc, H is Hex. F is Fucose. Cartoon symbols used for glycans (see inset) conform to the standard representation recommended by the Consortium for Functional Glycomics.

To investigate potential association between IRPC and protein glycosylation, we performed proteomics and *N*-glycosylation glycomics analysis (Tables S1-S5) in untreated LNCaP (NC), ST-EPI, LT-EPI, ST-ENZ, and LT-ENZ groups using Tandem Mass Tag (TMT) labels by nanoscale liquid chromatography coupled to tandem mass spectrometry (nanoLC-MS/MS). Pearson correlation coefficients between groups were all greater than 0.80, indicating a good reliability of experimental results (Fig. 2A). We identified 117 glycoproteins, 178 glycopeptides, 383 intact *N*-glycopeptides (IGPs), and 79 *N*-glycan (Fig. 2B & Tables S2-S5). The bulk of glycosylated peptides were of mannosylation (43%), followed by fucosylation (30%) in mono- (12.7%), di- (5.2%), tri- (7.8%) and ≥quadri-fucosylated (4.3%) form, representing 60% of all *N*-glycans identified (Fig. 2B).

Through quantitative analysis of glycoproteins, we found the level of growth/differentiation factor 15 (GDF15) glycoprotein was significantly upregulated in LT group, but not in ST group, compared to NC with both EPI and ENZ treatment (Fig. 2C). Increased GDF15 levels were reported to participate in the pathogenesis of metabolic diseases such as nonalcoholic fatty liver disease (NAFLD) and type 2 diabetes, cardiovascular disease such as hypertrophy and atherosclerosis, and some type of cancers such as PCa, breast cancer and gastric cancer [25–27]. Hence, we focused on the glycosylated GDF15 and its potential role in IRPC.

Subsequently we examined tryptic peptide glycotypes and 79 identified glycans were quantified. Levels of the majority of glycans, especially mannose-modified glycotypes, were downregulated, however, quantities of some complex glycan, such as N3H4F3, N6H3F2, N8H9F3S1 and N6H7S2, were upregulated in LT group compared to NC groups (Fig. 2D & Table S5). There were 13 glycoproteins modified by N3H4F3, 1 glycoprotein modified by N6H3F2, 3 glycoproteins modified by N8H9F3S1, and 2 glycoproteins modified by N6H7S2 (Fig. 2E). Among the 18 glycoproteins, only GDF15 was modified by two glycotypes, namely N3H4F3 and N6H3F2 (Fig. 2E). Because of the biological roles of glycan in cell signal transmission and molecular recognition, the two-glycan modified GDF15 as a glycosylated protein may play an essential role in the regulation of IRPC, which was further confirmed in the following described experiments.

### GDF15 is glycosylated at amino acid N70

**Fig. 3:**
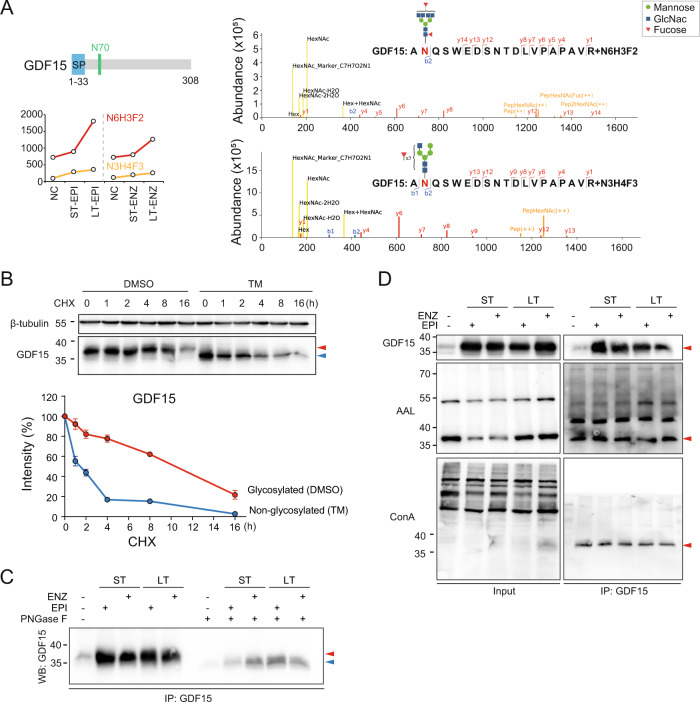
GDF15 is glycosylated at amino acid N70. **A** Levels of N6H3F2 and N3H4F3 glycan at GDF15 N70 are increased in CRPC cells. SP, signal peptide. Putative NQS motif in GDF15 is labeled in green. Numbers indicate amino-acid positions (left panel). MS spectrograms and fragment ion features of IGPs are shown (right panel). For each *N*-glycosylation site, one representative higher-energy collision dissociation (HCD) MS^2^ spectrum is shown to exemplify its identification based on detection of b and y ion (defining its peptide sequence by fracture mode of HCD). **B** Level of GDF15 protein is measured by WB in LNCaP cells in the presence or absence of 20 µm cycloheximide (CHX). Error bars indicate std. of biological triplicates. **C** GDF15 immunoprecipitants were treated with PNGase F and analyzed by WB. **D** Cell lysate (input) and GDF15 immunoprecipitant were used for lectin detection with Con A and AAL antibodies. Con A and AAL detect mannose and fucose, respectively. Red arrows point to glycosylated GDF15 and blue arrows point to non-glycosylated GDF15.

To explore the interaction between the core modified residue and complex glycans, the glycosylation pattern of GDF15 was detailed characterized with glycomics analysis. We found that both the N3H4F3 and N6H3F2 could attach to GDF15 N70, and N6H3F2 glycosylation was prominently upregulated in IRPC cells (Fig. 3A). In the presence of protein synthesis inhibitor cycloheximide (CHX), the turnover rate for non-glycosylated GDF15 was faster than their glycosylated ones. In addition, glycosylation of GDF15 was completely inhibited when cells were treated with the *N*-linked glycosylation inhibitor tunicamycin (TM) (Fig. 3B). To further validate the result, peptide-*N*-glycosidase F (PNGaseF) was employed. Glycans on glycosylated GDF15 (~38 kDa) was entirely removed, indicating GDF15 was modified by *N*-glycans (Fig. 3C). GDF15 immunoprecipitation and WB with Canavalia ensiformis (ConA) and Aleuria Aurantia Lectin (AAL) biotin antibody, which recognizes mannose and fucose respectively, indicated that GDF15 was modified by both mannose and fucose (Fig. 3D).

### PI3K-AKT pathway is enriched in the proteoglycomics dataset

**Fig. 4:**
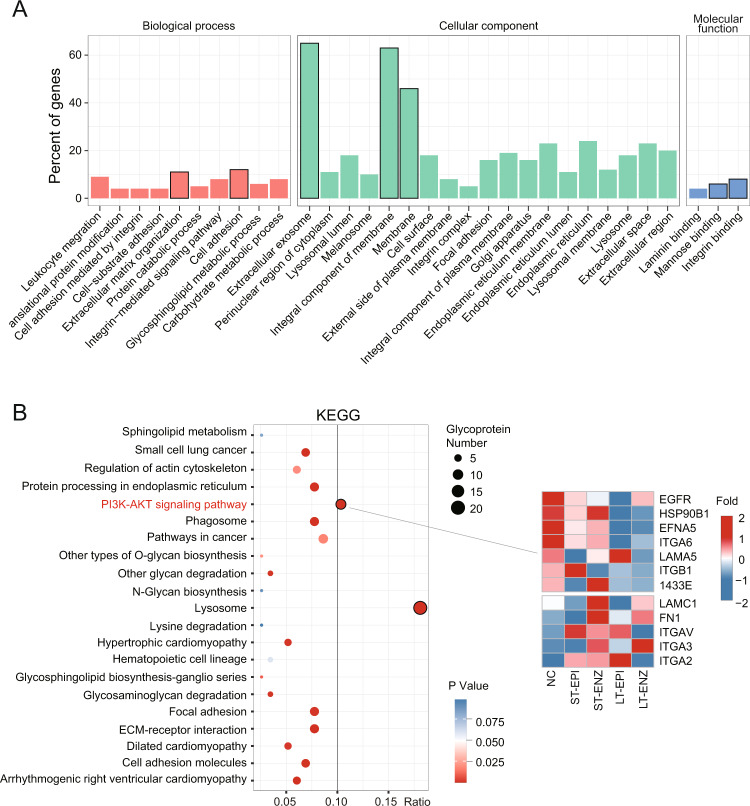
PI3K-AKT pathway is enriched in the proteoglycomics dataset. **A** Statistical distribution chart of differentially expressed glycoproteins under each GO category (2^nd^ Level). The *X*-axis label is divided into three parts: biological process, cellular component, and molecular function. **B** KEGG pathway enrichment bubble plot of differentially expressed glycoproteins (left panel). The size and color of the bubbles reflect the glycoprotein counts and *p* value, respectively. Heatmap of the PI3K-AKT pathway-related glycoprotein expression (right panel).

To explore potential mechanism involving in the development of IRPC, pathway enrichment analysis of GO and KEGG was performed using quantitative glycoproteins data (Table S2). We found that biological process enrichments were mainly related to cell adhesion and migration, that cellular component enrichments were in membrane and extracellular exosome, and that molecular function enrichments were mannose binding and integrin binding (Fig. 4A). Corresponding KEGG pathway analysis showed enrichments in lysosome and phosphatidylinositol 3-kinase (PI3K)-AKT signaling pathway (Fig. 4B). It is worth mentioning that the PI3K-AKT pathway including EGFR is reported to be activated in many human cancer types [28–31] and whether GDF15 acts as a suppressor or promoter related to EGFR expression in PCa remains unresolved [18, 26]. Thus, GDF15 glycosylation may influence drug-resistance through regulating its downstream PI3K-AKT pathway according to the above results and will further validated in Section 5.

### N70 glycosylation of GDF15 relieves its inhibitory effect on EGFR

**Fig. 5:**
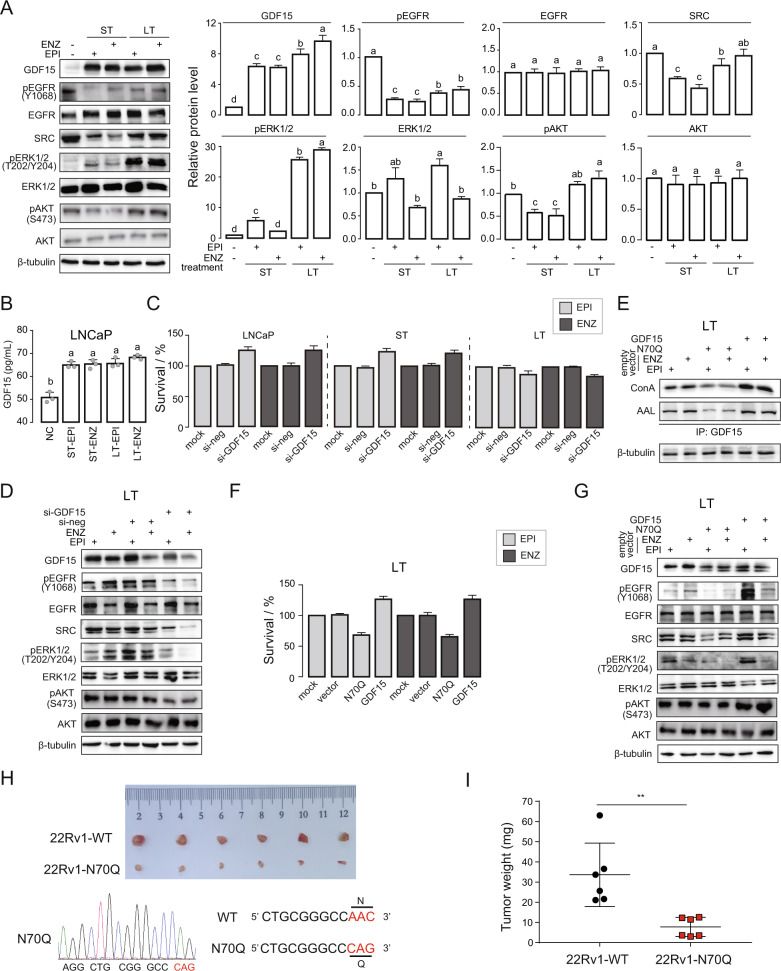
N70 glycosylation of GDF15 relieves its inhibitory effect on EGFR. **A** Levels of GDF15, pEGFR (Y1068), EGFR, SRC, pERK1/2 (T202/Y204), ERK1/2, pAKT (S473), and AKT proteins were measured by WB in NC, ST-EPI, ST-ENZ, LT-EPI, and LT-ENZ cells. β-tubulin protein was used as loading control. One-way ANOVA, *p* < 0.05. **B** Level of GDF15 in culture media was detected by ELISA in NC, ST-EPI, ST-ENZ, LT-EPI, and LT-ENZ cells. One-way ANOVA, *p* < 0.05. **C** Effect of GDF15 silencing on cells survival was detected by CCK8 in Parental, ST, and LT cells. **D** Levels of GDF15, pEGFR (Y1068), EGFR, SRC, pERK1/2 (T202/Y204), ERK1/2, pAKT (S473), and AKT proteins in corresponding samples were assessed by WB. **E** Lectin level was gauged by GDF15 immunoprecipitation-ConA and AAL WB in LT cells transfected with the wild-type GDF15 or N70Q mutant. **F** Cells survival rate was evaluated by CCK8 in corresponding treatments. **G** Levels of GDF15, pEGFR (Y1068), EGFR, SRC, pERK1/2 (T202/Y204), ERK1/2, pAKT (S473), and AKT proteins in corresponding samples were measured by WB. β-tubulin protein was utilized as loading control. **H** 22Rv1-WT (GDF15 overexpression) and 22Rv1-N70Q (GDF15 N70 site mutation) cells suspended in matrigel were injected into the right flank of BALB/c-nude mice. One week after injection, the tumor-bearing mice were castrated and following with tumor size monitoring. After 21 days, the mice were euthanized. The tumors were dissected, and representative images of six mice per group were illustrated (*n* = 6). Sanger sequencing results showed GDF15 with N70Q site mutation (AAC mutated into CAG) was successfully constructed in 22Rv1 cells. **I** The corresponding tumor weights were shown in the graph for clarity. Data represents the mean ± std. Student’s *t*-test, *p < 0.05.

It was reported that GDF15 could inhibit EGFR activity [18]. We surveyed the expression patterns of GDF15, pEGFR (Y1068), EGFR, SRC, pERK1/2 (T202/Y204), ERK1/2, pAKT (S473), and AKT proteins in NC, ST-EPI, ST-ENZ, LT-EPI, and LT-ENZ groups (Fig. 5A). AR inhibitors treatment increased total GDF15 protein in cells (Fig. 5A) as well as in culture medium (Fig. 5B), but EGFR protein level remained unchanged (Fig. 5A). Interestingly, pEGFR (Y1068) level was significant reduced in ST cells but partially recovered in LT cells, and SRC, pERK1/2 (T202/Y204), and pAKT (S473) levels changed accordingly (Fig. 5A). These results suggested that AR inhibitors may increase GDF15 level to block EGFR activation in ST, whereas such inhibitory effect is lost in LT treatment leading to drugs resistance.

To validate its role in regulating EGFR activity, we knocked down GDF15 in LNCaP, ST, and LT cells (Figure S1F, G) with siRNA. Silencing of GDF15 increased the survival of LNCaP and ST cells (Fig. 5C) along with the level of pEGFR (Y1068), SRC, pERK1/2 (T202/Y204), and pAKT (S473) (Figure S1H–I), whereas decreased the survival and the level of phosphoproteins in LT cells (Fig. 5D). Overexpression of the wild-type GDF15 increased GDF15 glycoprotein, pEGFR (Y1068), SRC, pERK1/2 (T202/Y204), and pAKT (S473) level along with cell survival (Fig. 5E–G). However, expression of N70Q, a glycosylation defective mutant, had opposite effect compared to the wild-type GDF15 (Fig. 5E–G). These data indicate that GDF15 inhibitory effect on EGFR and proliferation is lost in IRPC, and that the loss of GDF15 function is due to N70 site glycosylation.

To evaluate the role of GDF15 glycosylation in vivo, we used 22Rv1 cell (an ENZ-resistant cell line) in a xenograft model with male BALB/c nude mice castrated 21 days prior. Tumor growth in mice injected with cells expressing GDF15 N70Q mutant was significantly reduced compared to that of mice injected with control 22Rv1 cells (Fig. 5H, I). Thus, these results reveal that inhibiting GDF15 glycosylation at N70 site can effectively impede IRPC progression.

### SRC/AR inhibition significantly reduces tumor growth in castrated mice

**Fig. 6:**
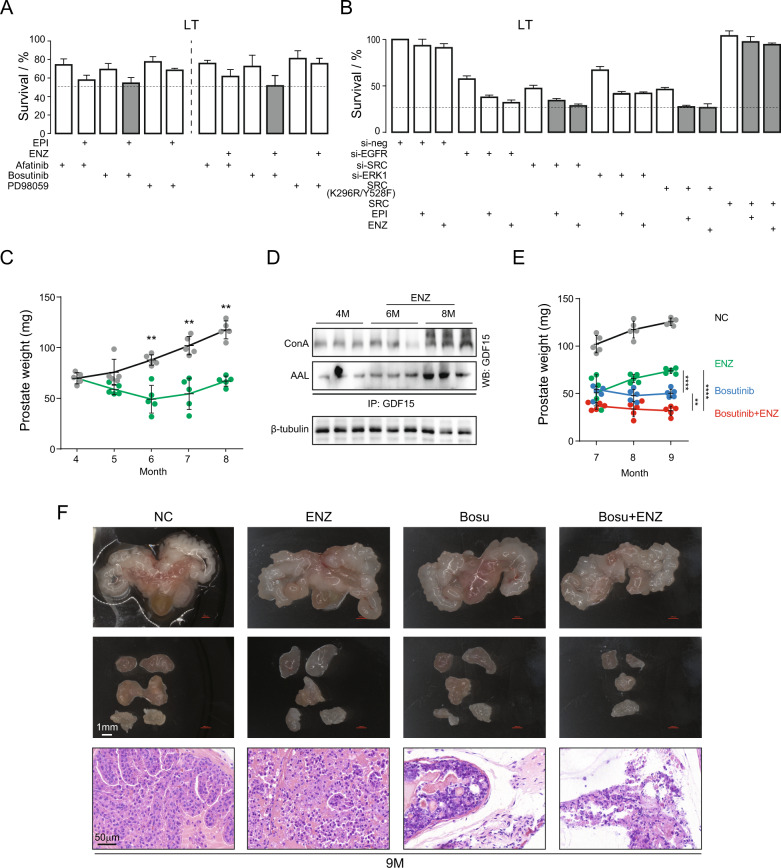
SRC/AR inhibition significantly reduces tumor growth in castrated mice. **A** LT cells were treated with Afatinib (EGFR inhibitor), Bosutinib (SRC inhibitor), and PD98059 (ERK inhibitor) or in combination with EPI or ENZ. **B** LT cells were treated with siRNAs (si-neg, si-EGFR, si-SRC, and si-ERK1), dominant negative SRC (DNsrc), and overexpression SRC or in combination with EPI or ENZ. Survival rates are shown. Error bars indicate mean ± std of biological triplicates. **C** Prostate weights in NC and ENZ treatment groups were shown in graph. 5 mice per group. Student’s *t*-test, *p < 0.05. **D** GDF15 was immunoprecipitated and then levels of mannose and fucose were quantified with ConA and AAL antibodies. β-tubulin protein was employed as input control. **E** Prostate weight was showed in graph. *n* = 5 mice per group. Student’s *t*-test, *p < 0.05. **F** Representative images of mouse prostate glands (up panel), prostate lobes (mid panel), and histopathological sections from NC, ENZ, Bosu, and Bosu + ENZ groups. NC negative control, ENZ Enzalutamide treatment, Bosu Bosutinib treatment, Bosu+ENZ, Bosutinib and Enzalutamide combined treatment. Data are expressed as mean ± std. of biological repetitions.

Since EGFR pathway is activated in IRPC, we treated LT cells with EGFR, SRC, ERK inhibitor or siRNA. Pathway interference significantly reduced LT cell survival, and together with AR inhibition further decreased survival rate (Fig. 6A, B). Inhibition of SRC seemed to be more effective than that of EGFR or ERK1. To corroborate this observation, we performed rescue experiments with the wild-type and kinase dead SRC. We found that overexpression of the wild-type SCR rescued the inhibition whereas overexpression of the dominant-negative SRC (K296R/Y528F) showed no relievable effect (Fig. 6B).

To further substantiate the therapeutic efficacy of SRC/AR dual targeting, we conducted in vivo experiments. AR inhibitor-resistant mice were generated using Hi-Myc mice [32] by continuous treatment with ENZ for three months. Prostate weights were reduced in the initial two-month exposure to AR inhibitor ENZ and increased starting at month three of the treatment, becoming castration-resistant (Fig. 6C). GDF15 glycosylation level was increased in CRPC tumors (Fig. 6D), which is consistent with LNCaP cell results. We then treated IRPC tumor with SRC inhibitor bosutinib. Bosutinib treatment alone effectively inhibited IRPC tumor growth, and treatment in combination with ENZ was significantly more efficacious (Fig. 6E, F). Inhibitor treatment, especially the bosutinib and ENZ combination, resulted in destruction of tumor cellular structure (Fig. 6F). Hence, in vivo results validate the in vitro observation.

### High level of GDF15 glycoprotein expression is associated with poor survival of cancer patients

**Fig. 7:**
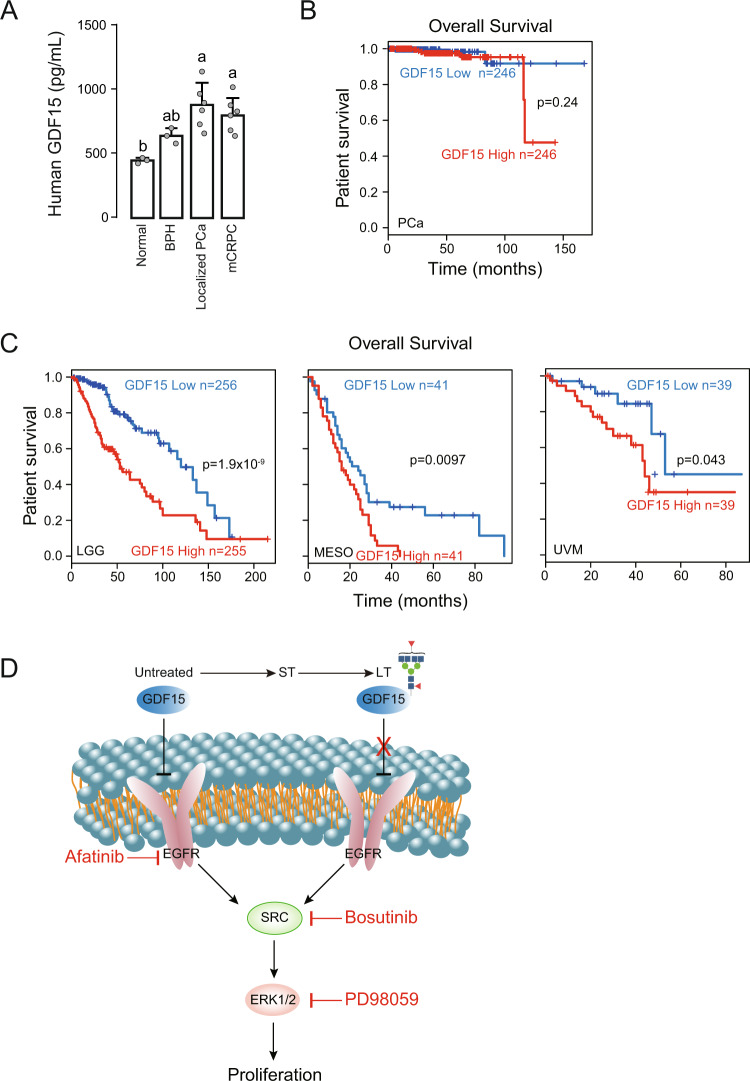
High level of GDF15 glycoprotein expression is associated with poor survival of cancer patients. **A** GDF15 level was determined by ELISA in serum samples from normal and patients with BPH, Localized PCa, or mCRPC. One-way ANOVA, *p* < 0.05. Data are expressed as mean ± std. **B** Prostate cancer patient overall survival curve in relation to GDF15 expression level was calculated using data from the Gene Expression Profiling Interactive Analysis (EGPIA) website. **C** Overall survival curves of lower grade glioma (LGG), mesothelioma (MESO), and uveal melanoma (UVM) patients in relation to GDF15 expression level were obtained from the EGPIA website. *P*-values were calculated using log-rank test; meta-analysis survival plots were generated using KM plotter. **D** Schematic graph depicts GDF15 inhibition of EGFR and N-glycosylation of GDF15 disables its suppressive effect on the EGFR pathway in CRPC cells.

To further support our observation, we investigated GDF15 expression in cancer patients. GDF15 level was significantly higher in the serum of patients with localized PCa and metastatic castration-resistant prostate cancer (mCRPC) (Fig. 7A). Although the overall survival of PCa patients was not notably correlated with GDF15 level, patients with high GDF15 expression had poor survival rate at the late stage of disease progression (Fig. 7B). Interestingly, the overall survivals of lower grade glioma (LGG), mesothelioma (MESO), and uveal melanoma (UVM) patients were significantly correlated with the GDF15 level (Fig. 7C).

Together, our study indicates that EGFR pathway is inhibited by GDF15 in untreated and short-term AR inhibitor treated LNCaP prostate cancer cells. Long-term treatment, however, induces GDF15 N70 glycosylation which relieves its inhibitory effect on EGFR, consequently EGFR activation contributes to the development of castration resistance (Fig. 7D).

## Discussion

Through proteomics and glycomics analyses, we identify GDF15 and show its regulation of EGFR pathway in PCa and importance in the development of AR inhibitor resistance. GDF15, a stress-induced cytokine, is known to have immunomodulatory functions and its high expression is often associated with cancer progression. However, whether GDF15 acts as a suppressor or promoter in prostate cancer remains unresolved [26]. Some evidence indicate that PCa induces osteocytes to secrete GDF15 stimulating tumor growth and invasion [33] and that over-expression of GDF15 leads to increased metastasis of PCa cells [34]. Inversely, expression of GDF15 in PCa cells inhibits cellular motility in vitro through a p53-dependent mechanism [35] and GDF15 is a mediator of nonsteroidal anti-inflammatory drugs (NSAIDs)-induced inhibition of migration of PCa cells [36]. We find that GDF15 level increases in the initial response of LNCaP cells to AR inhibitor treatment and loses its inhibitory effect on EGFR pathway during long-term AR blockade due to N-glycosylation. Our data suggest the importance of N70 glycosylation in regulating GDF15 function and may explain the controversial role of GDF15 in prostate cancer. PCa patients with high GDF15 expression have poor survival rate at the late stage of disease progression, which indicates GDF15, especially the corresponding glycol-modified form, may be a prognostic marker for late-stage prostate cancer.

Whether N70 glycosylation simply disables the ability of GDF15 to inhibit EGFR pathway or even stimulates EGFR signaling is currently unclear. It was reported that low-grade PCa had increased levels of paucimannosidic-(glycans with low percentage of mannose) and monoantennary-complex-type N-glycans compared to BPH, whereas high-grade PCa showed enrichment in highly branched complex-type N-glycans [14]. High mannose N-glycans reduce the contact area of cells with its substrate and may change the physical properties of cell membrane, promote migration of bone-marrow-derived mesenchymal stromal cells [37]. N6H3F2 (high mannose complex glycan) N-glycans possibly reduce the contact of GDF15 with EGFR, resulted in EGFR pathway activation. For instance, the core component of Hippo pathway (YAP) is O-GlcNAcylated by O-GlcNAc transferase (OGT) at S109, and YAP O-GlcNAcylation disrupts its interaction with upstream kinase LATS1, and activates its transcriptional activity [38]. Some glycosylation enzymes (GALNT7, GCNT1, TAP1, PGM3, *etc*.) are under the control of AR and link to the synthesis of cancer-associated glycans such as sialyl-Tn (sTn), sialyl LewisX (SleX) [39]. IRPC cells regain AR activity and would regulate glycosylation transferases, consequently GDF15 glycosylation. Therefore, how N70 glycosylation regulates GDF15 function warrants further investigation.

Although N6H3F2 modification of GDF15 may be a biomarker or therapeutic target for IRPC, selective drugs are not available. Methods targeting the GDF15 downstream pathway can be implemented. Breast cancer with high level of GDF15, especially in ErbB2 (HER2)-positive state, is sensitive to EGFR/ErbB2 inhibitor Lapatinib [40]. We show SRC inhibitor bosutinib, either alone or in combination with enzalutamide, is effective against IRPC. Clinical study of bosutinib in breast cancer (NCT00880009, NCT00959946) as EGFR pathway inhibitor or cyclin-dependent kinase (CDK) inhibitor have been implemented. Despite clinical trials show insignificant benefit in CRPC patients with EGFR inhibitors [22–24], AR and SRC dual-targeting for CRPC deserves exploring.

In conclusion, the present study uncovers a novel regulatory mechanism of IRPC, namely activation of the EGFR signaling pathway via N-glycosylation of GDF15 at the N70 site, demonstrates the validity of AR and SRC dual-targeting for IRPC, and lays foundation in the future development of selective inhibitors of GDF15 glycosylation for the management of CRPC.

## Methods

### Cell culture

LNCaP (ATCC) cell and 22Rv1 (ATCC) cell were grown in phenol red free Roswell Park Memorial Institute (RPMI)-1640 medium (Thermo Scientific, CA, USA) containing 5% fetal bovine serum (FBS; ThermoFisher), or 5% charcoal-stripped FBS (c-FBS; ThermoFisher) and 1% streptomycin-penicillin at 37 °C with 5% CO_2_. The cell lines were authenticated by short tandem repeat analysis and mycoplasma contamination was tested by the PCR Mycoplasma Detection Set (Takara, Otsu, Japan). LNCaP cell was treated with the ENZ (10 µM) or EPI (8 µM) at concentrations as reported [41, 42]. ST cell was treated for 9 days, and LT cell was treated for 33 days according to the method of Sharma et al. [43].

### Chemicals

Enzalutamide (MCE, HY-70002, NJ, US), EPI-001 (Selleck, S7955, TX, US), R1881 (Sigma, R0908, MO, US), Afatinib (Selleck, S1011, TX, US), Bosutinib (Selleck, S1014, TX, US), PD98059 (Selleck, S1177, TX, US), Cycloheximide (MCE, HY-12320, NJ, US), and Tunicamycin (MCE, HY-A0098, NJ, US) were stored as stock solutions in DMSO (Sigma, MO, US).

### Cell viability analysis

Cell viability was assessed by Cell Counting Kit (CCK-8; MCE, HY-K0301, NJ, US) according to the manufacturer’s instructions. Briefly, cells were seeded at a concentration of 6000 cells/200 µL/well into 96-well plates, incubated overnight, changed to fresh medium with various inhibitors. Following treatment, 10 µL CCK-8 solution were added and cells were incubated for 4 h at 37 °C. Optical density (OD) value was measured at 450 nm by a microplate spectrophotometer (Thermo Fisher). IC_50_ concentrations of ENZ and EPI were used in all drug treatment experiments. All experiments were performed three times in triplicates.

### Western blotting

Cells were treated as described and then lysed by boiling for 10 min in sample buffer (2% SDS, 10% glycerol, 10% β-mercaptoethanol, bromphenol blue and Tris-HCl, pH = 6.8). Lysates were fractionated on SDS-PAGE gels and transferred to PVDF membranes (Millipore, IPVH00010, NH, US). The blots were probed with specific antibodies followed by secondary antibody then membranes were detected by ECL (Sigma, WBULS0500, MO, US). AR (22089-1-AP; 1:1000), TMPRSS2 (14437-1-AP; 1:1000), PSA (60338-1-Ig; 1:2000), UBE2C (66087-1-Ig; 1:2000), CDC20 (10252-1-AP; 1:500), P21 (60214-1-Ig; 1:1000), Cyclin E1 (11554-1-AP; 1:1000), CDC6 (11640-1-AP; 1:2000), CDC2 (19532-1-AP; 1:1000), SRC (11097-1-AP; 1:500), GDF15 (27455-1-AP; 1: 2000), AKT (60203-2-Ig; 1: 5000), pAKT (66444-1-Ig; 4000), GAPDH (60004-1-Ig; 1:50000), β-actin (66009-1-Ig; 1:10000), β-tubulin (10068-1-AP; 1:4000) antibodies were purchased from Proteintech Group (IL, US). AR-V567es (ab200827; 1:1,000) antibodies were purchased from Abcam (MA, US). EGFR (4267; 1:2000), pEGFR (3777; 1:2000), ERK1/2 (9102; 1:2000), pERK1/2 (4376; 1:2000) antibodies were purchased from Cell Signaling Technology (MA, US). ConA (Canavalia ensiformis)-Biotin (C7401; 1:1000) antibody was purchased from Sigma (MO, US). AAL (Aleuria Aurantia Lectin)-Biotin (B-1395-1; 1:1000) antibody was purchased from Vector laboratories (CA, US).

Secondary antibodies were conjugated with HRP (Proteintech Group; SA00001-2, SA00001-1; 1:10000). Uncropped WB are shown in Figure S2.

### Cell cycle analysis

LNCaP-NC, ST, and LT cells were harvested and washed, and then fixed with 70% ethanol solution (v/v) at 4 °C for more than 18 h. After washing with pre-cold PBS, cells were stained with propidium iodide (PI) containing RNase (PI/RNase Staining Solution, CST 4087, MA, US) for 15 min in the dark, and then subjected to cell cycle analysis on a flow cytometer. The cell cycle data were analyzed using ModFit LT 5.0 software (Verity, ME, US).

### Proteomics study

LNCaP-NC, ST-EPI, ST-ENZ, LT-EPI, and LT-ENZ cells were used for quantitative proteomics analysis. Samples were lyzed with 8 M Urea (pH = 8.0) and concentration was quantified using BCA kit (Beyotime, P0012, Shanghai, China). Proteins were reduced with dithiothreitol (DTT) and then alkylated with iodoacetamide (IAM) in dark. Sequencing-grade trypsin (Promega, WI, USA) was added for overnight digestion. Peptides were desalted and reconstituted in 0.5 M tetraethyl-ammonium bromide (TEAB) and processed with TMT10 plex^TM^ kit according to the manufacturer’s protocol (Thermo Scientific, CA, USA). Global-peptides were resuspended in 2% acetonitrile (ACN) and 0.1% formic acid (FA) solution and then analyzed using an EASY-nLC 1200 system (Thermo Scientific, CA, USA) coupled with a high-resolution Orbitrap Fusion Lumos mass spectrum (Thermo Scientific). Peptides were first separated with an RSLC C18 column (1.9 µm × 100 µm × 20 cm) packed in house, then selected for MS/MS using NCE setting as 28, and the fragments were detected in the Orbitrap at a resolution of 17,500. A data-dependent procedure that alternated between one MS scan followed by 20 MS/MS scans with 15.0 s dynamic exclusion. Automatic gain control (AGC) was set at 5E4. Fixed first mass was set as 100 m/z.

### Glycomics study

Intact glycopeptides (IGPs) were enriched as described previously [44]. IGPs were desalted, resuspended and then analyzed using nanoLC-MS system. LC conditions and MS parameters for IGPs were described previously [44]. Results were filtered based on the following criteria: (1) a false discovery rate (FDR) less than 1% for glycoproteins and (2) each peptide spectra matches (PSM) annotated by at least one N-linked glycan.

### Glycoproteins and site analysis

The resulting MS/MS data were processed using Maxquant search engine (v.1.5.2.8). Tandem mass spectra were searched against homo_Uniprot-organism database (https://www.uniprot.org/taxonomy/9606) concatenated with reverse decoy database. For IGPs identification, data were searched using GPQuest 2.0 [45]. Parameters for analysis were described previously [44].

### Functional annotation and enrichment analysis

Gene Ontology (GO) annotation glycoproteome was derived from the UniProt-GOA database (https://www.uniprot.org/uniprot/A0A178W639). Firstly, identified protein IDs were converted to UniProt ID and then mapped to GO IDs by protein ID. If some identified proteins were not annotated by UniProt-GOA database, InterProScan (https://www.ebi.ac.uk/interpro/about/interproscan) would be used to annotated protein’s GO functional based on protein sequence alignment method. Then proteins were classified by Gene Ontology annotation based on three categories: biological process, cellular component, and molecular function. For enrichment of pathway analysis, Kyoto Encyclopedia of Genes and Genomes (KEGG) database (https://www.genome.jp/kegg/) was used to identify enriched pathways by a two-tailed Fisher’s exact test to detect the enrichment of differentially expressed protein against all quantitative glycoproteins. Pathways with a corrected FDR ≤ 0.05 were considered significant, and classified into hierarchical categories. Furthermore, proteins in selected pathways were visualized by a heat map using the “pheatmap” function from the “pheatmap” R-package (https://www.r-project.org/).

### Quantitative real-time PCR

LNCaP Cells were cultured with c-FBS for 5 days before treatment with DMSO + ENZ/EPI or R1881 + ENZ/EPI for 12 h. Total RNA was extracted using TRIzol (Takara, Japan) according to the manufacturer’s instructions. One µg of total RNA was used for complementary DNA synthesis using a cDNA reverse transcription kit (Takara, Japan). Real-time PCR was performed in triplicate using gene-specific primers on a Bio-Rad CFX96 PCR system. The gene-specific primers are listed in Table S7.

### Immunoprecipitation (IP)

Cells were lyzed in IP buffer (Beyotime, P0013, Shanghai, China). Protein samples were quantified using BCA kit (Beyotime, P0012, Shanghai, China) and precleared with A + G magnetic beads (MCE, HY-K0202, NJ, US). Immunoprecipitation was performed with GDF15 antibody (Abcam, ab206414, MA, US) overnight at 4 °C. Protein A + G magnetic beads were added, mixed for 4 h at 4 °C, captured with magnetic frame, washed 3 times, and then boiled in sample buffer. Supernatants were used for WB detection using GDF15, anti-Con A (Sigma, C7401) and AAL antibodies (Vector laboratories, B-1395-1).

### Enzyme-linked immunosorbent assay (ELISA)

GDF15 were quantified using human cell culture supernatant with ELISA kit from Mlbio (ml024335-2, Shanghai, China), according to the protocols provided by the manufacturers.

### Small-interfering RNA (siRNA)- mediated gene knockdown

EGFR siRNA (si-EGFR; 5′-CGCAAAGUGUGUAACGGAAUATTUAUUCCGUUACACACUUUGCGTT-3′), SRC siRNA (si-SRC; 5′-GACAGACCUGUCCUUCAAGAATTUUCUUGAAGGACAGGUCUGUCTT-3′), MAPK3 siRNA (si-ERK1; 5′-ACCUGCUGGACCGGAUGUUAATTUUAACAUCCGGUCCAGCAGGUTT-3′), and GDF15 siRNA (si-GDF15; 5′-CUAUGAUGACUUGUUAGCCAATTUUGGCUAACAAGUCAUCAUAGTT-3′) were made by Jiangsu Saisofi Biotechnology Co., Ltd (Wuxi, China), and Negative/GAPDH siRNA (si-neg; 5′-UUCUCCGAACGUGUCACG UTTACGUGACACGUUCGGAGAATT-3′/si-pos; 5′-UGACCUCAACUACAUGGUUTTAACCAUGUAGUUGAGGUCATT-3′) were used as negative/positive control. The siRNAs were transfected into Parental, ST, and LT cells using Polyplus-transfection (jetPRIME, NY, US) according to the manufacturer’s instructions. Successful knockdown was verified by quantitative real-time PCR (qRT-PCR) and WB.

### Plasmid- mediated overexpressing and gene mutation

Overexpression plasmids of pcDNA3.1(+)-GDF15 and pcDNA3.1(+)-SRC plasmids were constructed. Plasmids of pcDNA3.1(+)-GDF15/N70Q and dominant negative SRC (DCsrc)/K296R/Y528F were constructed. All plasmids were transiently transfected using Fugene HD transfection reagent (Promega, WI, USA).

### Animal study

For hypodermic CRPC xenografts, male BALB/c-nude mice (age of 8–9 weeks) were anaesthetized and then 1*10^6^ 22Rv1-WT or 22Rv1-N70Q stably transfected cells suspended in 30 µL 50% matrigel were surgically injected. One week after injection, the tumor-bearing mice were castrated and the tumor size was monitored weekly using vernier caliper. After 21 days, the mice were euthanized, tumors were dissected, photographed, and weighed. The experimental protocol was approved by the Animal Ethics Committee of Jiangnan University, China (JN.No20190630b2120101[190]).

Hi-Myc transgenic prostate cancer mice (gifted from George V. Thomas laboratory) [32] were used and all experimental protocols were approved by the Animal Ethics Committee of Jiangnan University, China (JN.No20190630t1360101[191]). To define temporal development of castration resistance, four-month-old male mice were randomly assigned to control (NC) and ENZ (10 mg/Kg) group, treated with drug by intragastric administration (i.g.) every 3 days for required months. The mice were then euthanized every month, and the prostate (anterior lobes, dorsal lateral lobes, and ventral lobes) were dissected, photographed, and weighed. After an initial decrease in prostate weight, regaining of prostate growth was considered as the emergence of castration resistance. Six-month old (4 months plus 2 months treatment) mice were randomly assigned to NC (saline solution i.g.), ENZ (10 mg/Kg i.g.), Bosutinib (10 mg/Kg i.g.), and Bosutinib + ENZ groups. Mice were treated every 3 days, euthanized every month. Prostate were dissected, photographed, weighed and then subjected to histopathological and WB analysis. Blood samples were collected from the orbital sinus and sera were separated, filtered, and stored at −80 °C until use.

### Tissue staining

Briefly, after deparaffinization and rehydration, 5 µm thick longitudinal sections were stained with hematoxylin solution for 22 s, dipped in 1% hydrochloric acid ethanol, rinsed with distilled water, stained with eosin solution for 30 s, dehydrated with graded alcohol and cleared in xylene. Mounted slides were scanned using an Pannoramic Scanner (3DHISTECH, Budapest, Hungary).

### Clinical study

Human peripheral blood samples were collected from the Affiliated Hospital of Jiangnan University. All patients signed informed consent. The study was approved and authorized by the Ethics Committee of the Affiliated Hospital of Jiangnan University (Approval document number: LS202128). The patients’ information is listed in Table S6.

### Statistical analysis

Student’s *t-test* was used to compare means of two groups. One-way ANOVA was used to compare means of 3 or more groups (GraphPad, CA, USA). Turkey test was used to perform multiple comparison (IBM SPSS, NY, USA). Data were presented as mean ± std of biological repetition. *P* < 0.05 was considered as significant in all of the tests.

## Supplementary information

checklist

Figure S1

Figure S2

Table S1

Table S2

Table S3

Table S4

Table S5

Table S6

Table S7

LNCaP-1

LNCaP-2

22Rv1-1

22Rv1-2

## Data Availability

All data supporting the findings of this study are available with the article, or from the corresponding author upon reasonable request. Data are also available via ProteomeXchange with identifier PXD030036.
