# Supplementary figures and images for: N-glycosylation of GDF15 abolishes its inhibitory effect on EGFR in AR inhibitor-resistant prostate cancer cells
Source: Cell Death Dis. 2022 Jul 19;13(7):626. doi: 10.1038/s41419-022-05090-3 (PMC9296468; doi:10.1038/s41419-022-05090-3)

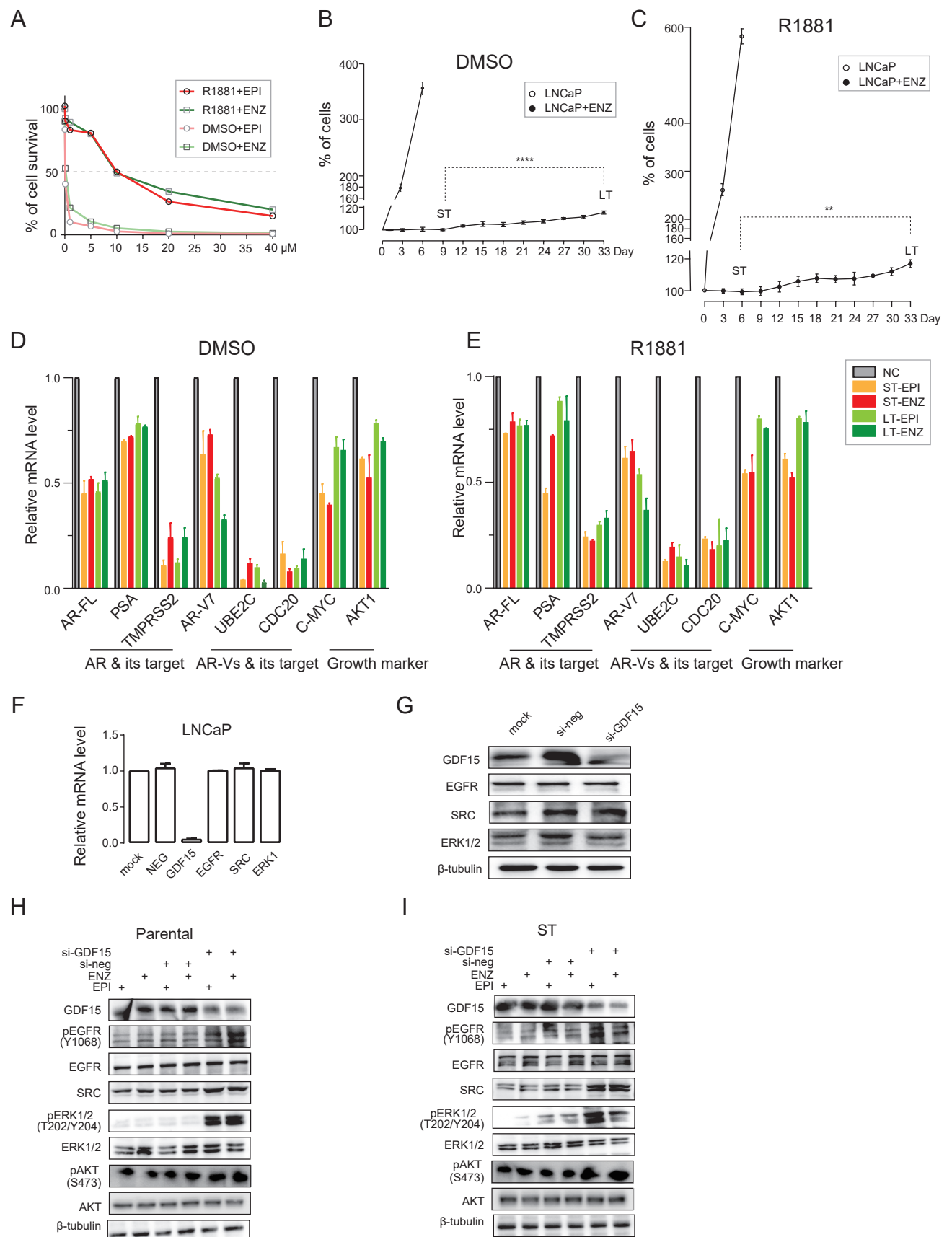

Figure S1

Supplement: Supplementary file 2 — Figure S1 [file 41419_2022_5090_MOESM2_ESM.pdf]
