# Supplementary material for: N-glycosylation of GDF15 abolishes its inhibitory effect on EGFR in AR inhibitor-resistant prostate cancer cells
Source: Cell Death Dis. 2022 Jul 19;13(7):626. doi: 10.1038/s41419-022-05090-3 (PMC9296468; doi:10.1038/s41419-022-05090-3)
Supplement: Supplementary file 3 — Figure S2 [file 41419_2022_5090_MOESM3_ESM.pdf]

Uncropped scans of the Western blots shown in the indicated figures

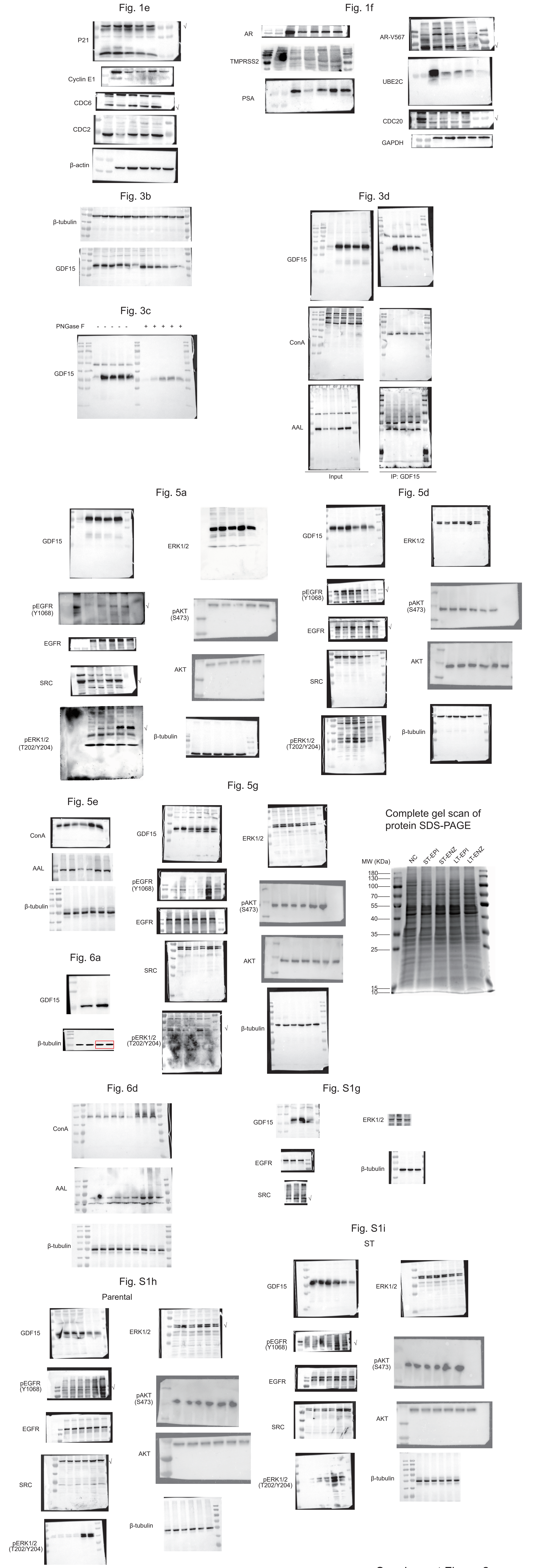

Supplement Figure 2
