# Supplementary material for: N-glycosylation of GDF15 abolishes its inhibitory effect on EGFR in AR inhibitor-resistant prostate cancer cells
Source: Cell Death Dis. 2022 Jul 19;13(7):626. doi: 10.1038/s41419-022-05090-3 (PMC9296468; doi:10.1038/s41419-022-05090-3)
Supplement: Supplementary file 9 — Table S6 [file 41419_2022_5090_MOESM9_ESM.docx]

Table S6. List of patient information

| Number | Diagnosis | Treatment | TPSA (ng/mL) | FPSA (ng/mL) |
| --- | --- | --- | --- | --- |
| 1 | N | / | <2 | / |
| 2 | N | / | <2 | / |
| 3 | N | / | <2 | / |
| 4 | BPH | / | 3.2 | / |
| 5 | BPH | / | 4.41 | 1.06 |
| 6 | BPH | / | <2 | / |
| 7 | LPCa | LRP | 12.17 | 1.3 |
| 8 | LPCa | LRP | 2.11 | 0.27 |
| 9 | LPCa | LRP | 6.86 | 0.66 |
| 10 | LPCa | LRP | 33.09 | 1.92 |
| 11 | LPCa | LRP | 26.94 | 1.86 |
| 12 | LPCa | LRP | 3.8 | 0.79 |
| 13 | CRPC | LRP+radiotherapy+DT | 13.3 | / |
| 14 | CRPC | LRP+DT | 31.81 | / |
| 15 | CRPC | LRP+chemotherapy+castrate+DT | 1.33 | / |
| 16 | CRPC | LRP+DT+radiotherapy | 56.23 | / |
| 17 | CRPC | LRP+DT | >154 | / |
| 18 | CRPC | LRP+chemotherapy+castrate+DT | 3.35 | / |

N, Normal; BPH, Benign Prostatic Hyperplasia; LPCa, Localized Prostate Cancer; CRPC, Castration-Resistant Prostate Cancer; LRP, Laparoscopic Radical Prostatectomy; DT, Drug Treatment.
