# Supplementary material for: N-glycosylation of GDF15 abolishes its inhibitory effect on EGFR in AR inhibitor-resistant prostate cancer cells
Source: Cell Death Dis. 2022 Jul 19;13(7):626. doi: 10.1038/s41419-022-05090-3 (PMC9296468; doi:10.1038/s41419-022-05090-3)
Supplement: Supplementary file 10 — Table S7 [file 41419_2022_5090_MOESM10_ESM.docx]

Table S7. Sequence of quantitative-PCR primers

| Name of Gene | Primer (5’ to 3’) |
| --- | --- |
| *GDF15-F* | GACCCTCAGAGTTGCACTCC |
| *GDF15-R* | GCCTGGTTAGCAGGTCCTC |
| *EGFR-F* | AGGCACGAGTAACAAGCTCAC |
| *EGFR-R* | ATGAGGACATAACCAGCCACC |
| *SRC-F* | TGGCAAGATCACCAGACGG |
| *SRC-R* | GGCACCTTTCGTGGTCTCAC |
| *ERK1-F* | CTACACGCAGTTGCAGTACAT |
| *ERK1-R* | CAGCAGGATCTGGATCTCCC |
| *AR-FL-F* | ACATCAAGGAACTCGATCGTATCATTGC |
| *AR-FL-R* | TTGGGCACTTGCACAGAGAT |
| *PSA-F* | GGTGACCAAGTTCATGCTGTG |
| *PSA-R* | GTGTCCTTGATCCACTTCCG |
| *TMPRSS2-F* | CTGGTGGCTGATAGGGGATA |
| *TMPRSS2-R* | GGACAAGGGGTTAGGGAGAG |
| *AR-V7-F* | CCATCTTGTCGTCTTCGGAAATGTTATGAAGC |
| *AR-V7-R* | TTTGAATGAGGCAAGTCAGCCTTTCT |
| *UBE2C-F* | TGGTCTGCCCTGTATGATGT |
| *UBE2C-R* | AAAAGCTGTGGGGTTTTTCC |
| *CDC20-F* | CGGAAGACCTGCCGTTACATTC |
| *CDC20-R* | CAGAGCTTGCACTCCACAGGTA |
| *C-MYC-F* | CCTGGTGCTCCATGAGGAGAC |
| *C-MYC-R* | CAGACTCTGACCTTTTGCCAGG |
| *AKT1-F* | TGGACTACCTGCACTCGGAGAA |
| *AKT1-R* | GTGCCGCAAAAGGTCTTCATGG |
| *GAPDH-F* | ACCCAGAAGACTGTGGATGG |
| *GAPDH-R* | TTCAGCTCAGGGATGACCTT |
