# Supplementary material for: N-glycosylation of GDF15 abolishes its inhibitory effect on EGFR in AR inhibitor-resistant prostate cancer cells
Source: Cell Death Dis. 2022 Jul 19;13(7):626. doi: 10.1038/s41419-022-05090-3 (PMC9296468; doi:10.1038/s41419-022-05090-3)
Supplement: Supplementary file 11 — LNCaP-1 [file 41419_2022_5090_MOESM11_ESM.pdf]

STR 细胞鉴定结题报告

订单编号: F21FTSCCLH0036

报告时间: 2021.06.11

目录

1 实验目的 3

2 实验流程 3

2.1 实验试剂 3

2.2 实验仪器 3

2.3 实验步骤 3

2.4 上机检测 3

3 实验结果 4

3.1 LNCaP STR 数据 4

3.2 LNCaP STR 位点数据比对结果 5

3.3 LNCaP STR 图谱 5

4. 参考文献 6

1 实验目的

细胞 STR 信息鉴定：LNCaP clone FGC (人前列腺癌细胞)

2 实验流程

2.1 实验试剂

人类 DNA 分型盒 （炎黄）

2.2 实验仪器

GeneAmp® PCR system9700，ABI3730XL

2.3 实验步骤

DNA 提取后，按以下体系进行反应扩增

ddH2O	2	μl
2X Master Mix	5	μl
4X Primer Pair Mix	2	μl
DNA	1	μl
Total	10	μl

PCR 扩增使用 Applied Biosystems 9700 PCR System 进行扩增，PCR 扩增程序如下：

95℃	10min	
95℃	10 sec	×28Cycle
58℃	1min	×28Cycle
70℃	20 sec	×28Cycle
60℃	15 min	
4℃	∞	

2.4 上机检测

内标混合：将 PCR 产物与 STR 500 内标，HIDI 混合，混合体系为

STR-500	0.5μl
PCR 产物	1μl
HIDI	8.5μL
Total	10μL

将混合物在 PCR 仪上进行热变性（95℃3min40s），冰中骤冷，待上机。

使用仪器为 3730XL 按仪器操作说明书安装毛细管，进行毛细管位置的校正，人工手动灌胶和建立运行的测序文件。仪器将自动灌胶至毛细管，1.2kV 预电泳 5min，按编程次序自动进样，再预电泳（1.2kV，20min），在 7.5kV 下电泳 2h。电泳结束后仪器会自动清洗，灌胶，进下一样品，预电泳和电泳。每一个样品电泳总时间为 2.5h。电泳结束后仪器会自动分析 STR 图谱。

3 实验结果

3.1 LNCaP STR 数据

STR 基因座	LNCaP 检测结果			
	Allele 1	Allele 2	Allele 3	Allele 4
Yindel	2			
AMEL	X	Y		
D3S1358	16			
D13S317	10	12		
D7S820	9.1	10.3		
D16S539	11			
SE33	16			
D10S1248	14	16		
D5S818	11	12		
D21S11	29	32.2		
TPOX	8	9		
D1S1656	16			
D6S1043	11	12	18	
DXS6795	9			
D19S433	13.2	15		
D22S1045	15	17		
D8S1179	11	12	13	14
Penta E	12	16		
DYS391	9	10		
D2S441	11.3			
D12S391	21	22		
D2S1338	16			
vWA	16	18		
Penta D	12	12.4		
TH01	9			
D18S51	11	12		
CSF1PO	10	11		
FGA	19	20		

3.2 LNCaP STR 位点数据比对结果

通过 DSMZ 数据库/ATCC数据库/客户提供细胞信息比对结果如下

EV	Cell No.	Cell name	Locus names								
			D5S818	D13S317	D7S820	D16S539	VWA	TH01	AM	TPOX	CSF1PO
	Query (Your Cell)		11,12	10,12	9.1,10.3	11,11	16,18	9,9	X,Y	8,9	10,11
1.00(36/36)	256	LNCAP	11,12	10,12	9.1,10.3	11,11	16,18	9,9	X,Y	8,9	10,11

通过数据库比对，LNCaP 样本的 STR 结果符合数据库的 STR 信息。

注：参考 ANSI/ATCC,Authenticationof Human Cell Line Standardization of STR Profiling . 2011,ASN-0002-2011.标准，STR 检测匹配度超过 80%即可认为是同一种来源的细胞。

3.3 LNCaP STR 图谱

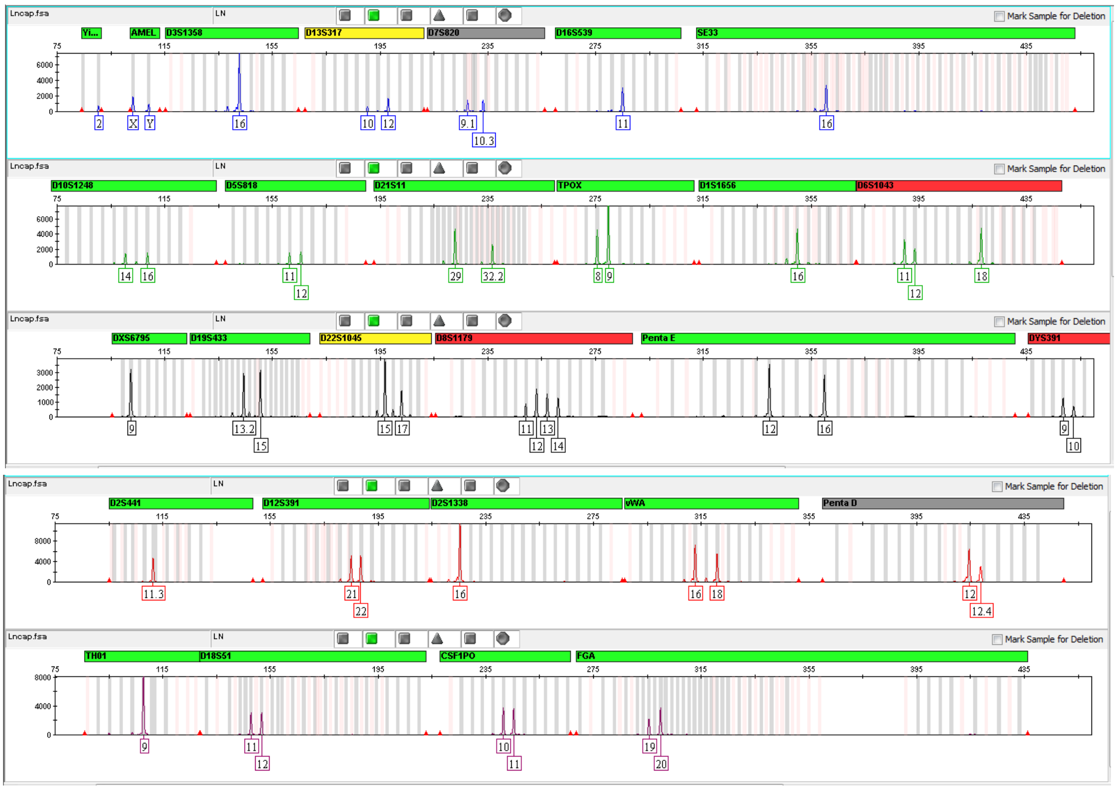

4. 参考文献

1 Zhao, M., et al., Assembly and initial characterization of a panel of 85 genomically validated cell lines from diverse head and neck tumor sites. Clin Cancer Res, 2011. 17(23): p. 7248-64.

2 Masters, J.R., Cell-line authentication: End the scandal of false cell lines. Nature, 2012. 492(7428): p. 186.

3 American Type Culture Collection Standards Development Organization Workgroup, A.S.N., Authentication of Human Cell Lines: Standardization of STR Profiling. 2011, ANSI/ATCC ASN-0002-2011.

4 Reid, Y.A., Characterization and authentication of cancer cell lines: an overview. Methods Mol Biol, 2011. 731: p. 35-43.

5 Lorsch, J.R., F.S. Collins, and J. Lippincott-Schwartz, Cell Biology. Fixing problems with cell lines. Science, 2014. 346(6216): p. 1452-3.

6 Chatterjee, R., Cell biology. Cases of mistaken identity. Science, 2007. 315(5814): p. 928-31.
