# Supplementary material for: N-glycosylation of GDF15 abolishes its inhibitory effect on EGFR in AR inhibitor-resistant prostate cancer cells
Source: Cell Death Dis. 2022 Jul 19;13(7):626. doi: 10.1038/s41419-022-05090-3 (PMC9296468; doi:10.1038/s41419-022-05090-3)
Supplement: Supplementary file 12 — LNCaP-2 [file 41419_2022_5090_MOESM12_ESM.pdf]

LNCaP（人前列腺癌细胞）支原体检测实验操作报告

1 主要仪器试剂

1.1 主要仪器

表 1.1 实验所用主要仪器

Main instruments for the experiment

所用仪器	仪器型号	公司
全功能微孔板检测仪	PerkinElmer/envision	PerkinElmer/envision
冷冻离心机	neofuge 15R	Heal force
电热恒温水浴锅	HH-US-A	美标
Aquapro 超级纯水仪	艾科浦	AJY -0501
PCR 仪	东胜创新生物科技有限公司	EDC-810
水平电泳仪	北京君意东方电泳设备有限公司	JY300
电子天平	北京赛多利斯仪器系统有限公司	CPA

1.2 主要试剂

支原体 PCR 检测试剂盒

2 实验内容

2.1 实验流程

细胞沸水浴 10min

↓

12000rpm 离心 3-5 分钟

↓

吸取 4ul 上清液进行 PCR

↓

配制 2%琼脂胶电泳

↓

得到胶图　　　　　　　　↓

2.2 实验操作

2.2.1 实验步骤

（1）将细胞沉淀及上清混匀，进行沸水浴10min。

（2）12000rp离心3-5min后取上清备用。

（3）配制PCR反应体系，如下表：

成分	阴性对照	阳性对照	实验组
PCR 反应液	14ul	14ul	14ul
引物	2ul	2ul	2ul
阳性对照	4ul	-	-
待测样品	-	-	4ul
H_2O	4ul	-	-

反应程序如下：

94℃ 5min

30cycle { 94℃ 130s; 55℃ 30s; 72℃ 15s }

94℃　5min

(4) 琼脂糖凝胶电泳。

(5) 凝胶成像分析。

3. 实验结果与分析

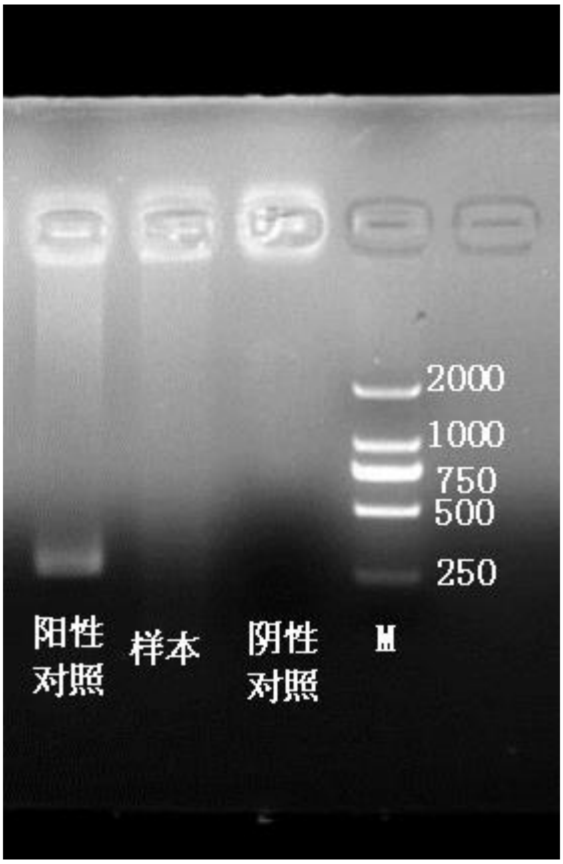

附图：支原体检测凝胶图谱

电泳图检测结果显示：

① 实验中阳性对照有条带，阴性对照无条带，证明本次实验结果准确可靠。

② 样本 LNCaP（人前列腺癌细胞）无条带，为支原体阴性。

（此结果仅对本次检材负责）

操作人：明林峰　　　　　　　　　　　　审核人：敖章恒

检测日期：2022.03.10
