# Supplementary material for: N-glycosylation of GDF15 abolishes its inhibitory effect on EGFR in AR inhibitor-resistant prostate cancer cells
Source: Cell Death Dis. 2022 Jul 19;13(7):626. doi: 10.1038/s41419-022-05090-3 (PMC9296468; doi:10.1038/s41419-022-05090-3)
Supplement: Supplementary file 13 — 22Rv1-1 [file 41419_2022_5090_MOESM13_ESM.pdf]

STR 细胞鉴定结题报告

订单编号： F21FTSCCLH0006

报告时间： 2021.01.29

目录

1 实验目的 3

2 实验流程 3

2.1 实验试剂 3

2.2 实验仪器 3

2.3 实验步骤 3

2.4 上机检测 3

3 实验结果 4

3.1 22RV1 STR 数据 4

3.2 22RV1 STR 位点数据比对结果 5

3.3 22RV1 STR 图谱 5

4. 参考文献 6

1 实验目的

细胞STR 信息鉴定：22RV1 人前列腺癌细胞

2 实验流程

2.1 实验试剂

Investigator® 24plex GO!Kit (200)

2.2 实验仪器

GeneAmp® PCR system9700，ABI3730XL

2.3 实验步骤

DNA 提取后，按以下体系进行反应扩增

ddH2O	2	µl
2X Master Mix	5	µl
4X Primer Pair Mix	2	µl
DNA	1	µl
Total	10	µl

PCR 扩增使用Applied Biosystems 9700 PCR System 进行扩增，PCR 扩增程序如下：

95℃	10 min	
95℃	10 sec	×28Cycle
58℃	1min	×28Cycle
70℃	20 sec	×28Cycle
60℃	15min	
4℃	∞	

2.4 上机检测

内标混合：将PCR 产物与STR 500 内标，HIDI 混合，混合体系为

STR-500	0.5µl
PCR 产物	1µl
HIDI	8.5µL
Total	10µL

将混合物在PCR 仪上进行热变性（95℃3min40s），冰中骤冷，待上机。

使用仪器为 3730XL 按仪器操作说明书安装毛细管，进行毛细管位置的校正，人工手动灌胶和建立运行的测序文件。仪器将自动灌胶至毛细管，1.2kV 预电泳 5min，按编程次序自动进样，再预电泳（1.2kV，20min），在 7.5kV 下电泳 2h。电泳结束后仪器会自动清洗， 灌胶，进下一样品，预电泳和电泳。每一个样品电泳总时间为 2.5h。电泳结束后仪器会自动分析STR 图谱。

3 实验结果

3.1 22RV1 STR 数据

STR 基因座	22RV1 检测结果		
	Allele 1	Allele 2	Allele 3
AM	X	Y	
TH01	6	9.3	
D3S1358	15		
vWA	15	21	
D21S11	30		
TPOX	8		
DYS391	11		
D1S1656	17	18	19
D12S391	18	25	
SE33	24.2	30.2	31.2
D10S1248	13	14	
D22S1045	15		
D19S433	13	14	
D8S1179	12	13	14
D2S1338	17	18	
D2S441	10	12	
D18S51	13	14	
FGA	20	23	
QS1	Q		
D16S539	12		
CSF1PO	10	11	
D13S317	8	12	
D5S818	11	13	
D7S820	9	10	11
QS2	S		

3.2 22RV1 STR 位点数据比对结果

通过DSMZ 数据库/ATCC数据库/客户提供细胞信息比对结果如下

EV	Cell No.	Cell name	Locus names								
			D5S818	D13S317	D7S820	D16S539	VWA	TH01	AM	TPOX	CSF1PO
	Query (Your Cell)		11,13	8,12	9,10,11	12,12	15,21	6,9.3	X,Y	8,8	10,11
0.92(36/39)	438	22RV1	11,12,13	9,12	10,11,9	12,12	15,21	6,9.3	X,Y	8,8	10,11
0.92(36/39)	CRL-2505	22Rv1	11,12,13	9,12	9,10,11	12,12	15,21	6,9.3	X,Y	8,8	10,11

<u>通过数据库比对，22RV1 样本的 STR 结果符合数据库的 STR 信息。</u>

注：参考 ANSI/ATCC,Authenticationof Human Cell Line Standardization of STR Profiling . 2011,ASN-0002-2011.标准，STR 检测匹配度超过 80%即可认为是同一种来源的细胞。

3.3 22RV1 STR 图谱

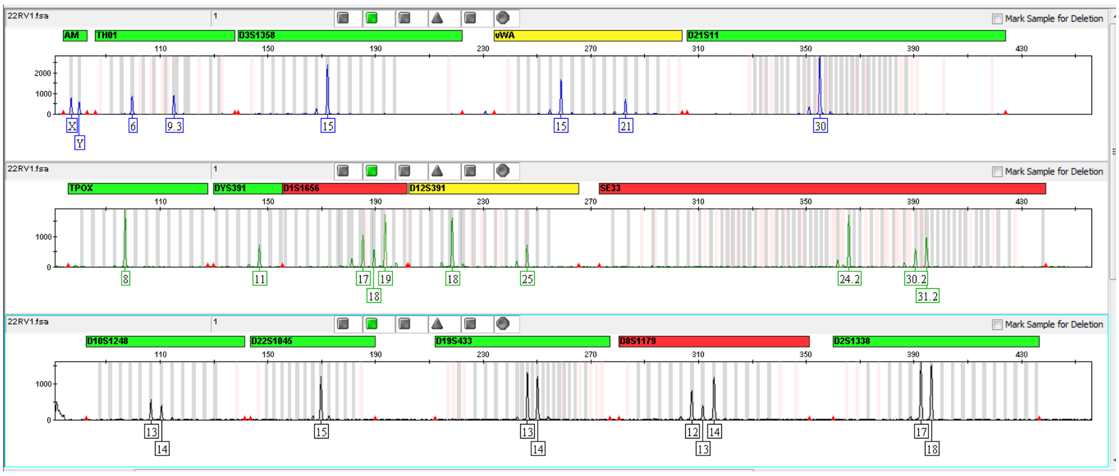

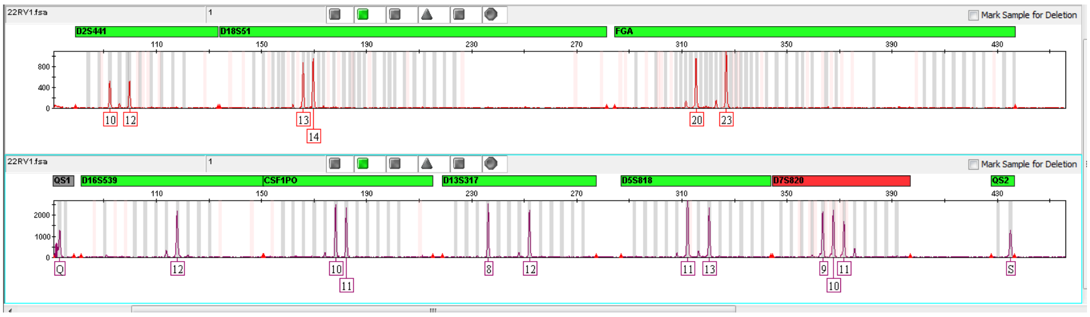

4. 参考文献

1 Zhao, M., et al., Assembly and initial characterization of a panel of 85 genomically validated cell lines from diverse head and neck tumor sites. Clin Cancer Res, 2011. 17(23): p. 7248-64.

2 Masters, J.R., Cell-line authentication: End the scandal of false cell lines. Nature, 2012. 492(7428): p. 186.

3 American Type Culture Collection Standards Development Organization Workgroup, A.S.N., Authentication of Human Cell Lines: Standardization of STR Profiling. 2011, ANSI/ATCC ASN-0002-2011.

4 Reid, Y.A., Characterization and authentication of cancer cell lines: an overview. Methods Mol Biol, 2011. 731: p. 35-43.

5 Lorsch, J.R., F.S. Collins, and J. Lippincott-Schwartz, Cell Biology. Fixing problems with cell lines. Science, 2014. 346(6216): p. 1452-3.

6 Chatterjee, R., Cell biology. Cases of mistaken identity. Science, 2007. 315(5814): p. 928-31.
